# Precise Monitoring of Soil Salinity in China’s Yellow River Delta Using UAV-Borne Multispectral Imagery and a Soil Salinity Retrieval Index

**DOI:** 10.3390/s22020546

**Published:** 2022-01-11

**Authors:** Xinyang Yu, Chunyan Chang, Jiaxuan Song, Yuping Zhuge, Ailing Wang

**Affiliations:** 1 College of Resources and Environment, Shandong Agricultural University, Tai’an 271018, China; yuxy.12b@igsnrr.ac.cn (X.Y.); chyan0103@sdau.edu.cn (C.C.); SJxuan020903@163.com (J.S.); ailingwang@sdau.edu.cn (A.W.); 2 Tropical Research and Education Center/Department of Agricultural and Biological Engineering, Institute of Food and Agricultural Sciences, University of Florida, Homestead, FL 33031, USA

**Keywords:** soil salinity sensitive parameter, random forest, support vector machine, optimal retrieval model, remote sensing

## Abstract

Monitoring salinity information of salinized soil efficiently and precisely using the unmanned aerial vehicle (UAV) is critical for the rational use and sustainable development of arable land resources. The sensitive parameter and a precise retrieval method of soil salinity, however, remain unknown. This study strived to explore the sensitive parameter and construct an optimal method for retrieving soil salinity. The UAV-borne multispectral image in China’s Yellow River Delta was acquired to extract band reflectance, compute vegetation indexes and soil salinity indexes. Soil samples collected from 120 different study sites were used for laboratory salt content measurements. Grey correlation analysis and Pearson correlation coefficient methods were employed to screen sensitive band reflectance and indexes. A new soil salinity retrieval index (SSRI) was then proposed based on the screened sensitive reflectance. The Partial Least Squares Regression (PLSR), Multivariable Linear Regression (MLR), Back Propagation Neural Network (BPNN), Support Vector Machine (SVM), and Random Forest (RF) methods were employed to construct retrieval models based on the sensitive indexes. The results found that green, red, and near-infrared (NIR) bands were sensitive to soil salinity, which can be used to build SSRI. The SSRI-based RF method was the optimal method for accurately retrieving the soil salinity. Its modeling determination coefficient (*R^2^*) and Root Mean Square Error (*RMSE*) were 0.724 and 1.764, respectively; and the validation *R^2^*, *RMSE*, and Residual Predictive Deviation (*RPD*) were 0.745, 1.879, and 2.211.

## 1. Introduction

Soil is a vital component of the ecosystem. It plays a crucial role in the structure and operation of the land ecosystem [1,2]. However, the degradation of soil resources has emerged as one of the world’s most pressing ecological concerns. Soil salinization has already become a significant symptom of soil degradation that affects 10% of the world’s agricultural land [3,4]. The search for a reliable monitoring index and precise regression method for soil salinity is essential to globally assess soil salinization and its severe implications for agriculture and food security.

Ecological parameter measurement and airborne/satellite remote sensing (RS) monitoring technologies are two commonly utilized soil salinity assessment methods. Traditional methods rely on field surveys and electrical conductivity measurements, which are accurate but time and labor-intensive [5,6], and do not allow for monitoring of the spatial distribution pattern of soil salinity content. Multi- and hyperspectral satellite RS technology has been used in soil salinity monitoring since the 1990s [7,8]. Azabdaftari et al. (2016), for instance, computed vegetation indexes in the Adana region of Turkey using Landsat multispectral images from four different times [9]. Morgan et al. (2018) forecasted soil salinity in Cairo, Egypt using Sentinel-2 multispectral data [10]. Hyperspectral images such as EO-1 and HJ-1A were also employed as data sources to accurately detect soil salinity [11,12]. Different from the satellite RS means, the Unmanned Aerial Vehicle (UAV)-borne spectral sensors are highly maneuverable and have been used to monitor soil salinity since the 2010s. Hu et al. (2019) used electromagnetic induction equipment and a hyperspectral camera mounted on a UAV platform to evaluate and estimate field-scale soil salinity [13]. Ivushkin (2019) looked into the use of UAVs to measure salt stress in quinoa plants [14]. Wang et al. (2019) extracted the salt content of extremely salty soil in China’s Yellow River Estuary and compared the retrieval findings with the inverse distance weighted interpolation results to achieve more accurate saline soil extraction [15]. To boost the spectral resolution to retrieve soil salinity, Ma (2020) combined Sentinel-2A and UAV multispectral images to increase the spectral resolution to inverse regional soil salinity [16]. Satellite RS imagery-based soil salinity studies have indicated that the index in the visible to infrared spectrum may better measure soil salinity, which can increase the accuracy of soil salinity retrieval [17,18,19]. The majority of vegetation indexes can indirectly indicate soil salinity [20]. However, few studies focused on the detection of UAV band information sensitive to soil salinity, which is essential for the construction of a reliable soil salinity monitoring index to help efficiently predict the soil salinity conditions.

For the soil salinity regression method, several approaches such as partial least square (PLS), BP Neural Network (BPNN), Support Vector Machines (SVM), and random forest (RF) were introduced and applied [15,21]. For instance, Ma (2018) increased the accuracy of soil salinization retrieval by combining numerous mathematical changes on soil surface reflectance with regression analysis of collected soil data [12]. Machine learning algorithms were used by Yao et al. (2019) to infer agriculture soil salt concentration from UAV multispectral RS images [22]. The determination coefficients for validation were more than 0.69. To improve regional retrieval precision, Chen et al. (2021) presented a differentiated fusion method for calculating satellite and ground spectral variables of soil salinity based on sample differences [23]. Spectral parameters and correlation salinity indexes have been converted and filtered to retrieve soil salinity. In resource management and allocation, the river delta region has a high degree of social-ecological interdependence and competition. In China, the Yellow River Delta (YRD) features shallow groundwater levels (0–2 m), significant salinity, and surface salinity. Soil salinization affects over 70% of YRD’s land, making the region’s biological ecosystem severely vulnerable [24]. Soil salinization has long been a major source of soil degradation in the YRD, limiting local agricultural productivity. Precise monitoring of soil salinity is essential to assess soil salinization. However, screening and design of sensitive parameters, as well as a suitable retrieval method, is, nevertheless, unknown.

This study thus strived to explore the sensitive parameter and construct an optimal method for soil salinity retrieval. The Yellow River Delta (YRD) in China was selected as the study area to experiment. UAV RS image and ground truth data collected during the spring season were used as the data source. Sensitive bands and spectral parameters of soil salinity were identified using grey correlation analysis and Pearson correlation coefficient approaches. PLSR, MLR, BPNN, SVM, and RF modeling methods were used to create soil salt retrieval models based on reflectance, vegetation index, and salinity index. The accuracies were evaluated quantitatively to find the optimal retrieval model. This study is expected to serve as a guide for the selection of sensitive criteria and the optimal soil salinity prediction algorithms, which can be used in other regions to retrieve soil salinity efficiently.

## 2. Materials and Methods

### 2.1. Study Area

The study was conducted in a representative arable region of Kenli District, YRD (37°35′6”~37°35′14′ N, 118°20′31″~118°20′46″ E). The climate of the study area is a temperate continental monsoon climate, which is dry and windy in spring. With a potential evapotranspiration–precipitation ratio of 7.6, potential evapotranspiration considerably outnumbers precipitation in spring, resulting in limited vegetation covering in the study area and severe salt deposition in the soil. The groundwater table is also shallow and mineralized. Arable and abandoned lands are the most common land uses, and coastal (tidal) salty soil with a light texture and high capillary action is the most common soil type. Hydrogeological conditions in the study area may contribute to soil salinization [25].

### 2.2. Image Acquisition and Preprocessing

**Figure 1:**
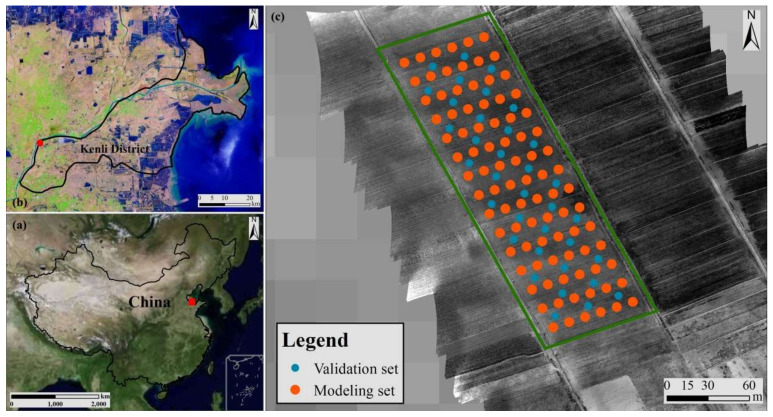
Location of the study area. (**a**) Location of the Kenli District in China; (**b**) test area in the Kenli district; (**c**) UAV image covering the test area.

The spring season in the study area is the period of high evapotranspiration and accumulation of soil salinity, which is crucial for the development of winter wheat. On 19 March 2021, a field survey was conducted to collect soil samples and obtain UAV images (Figure 1). The DJI Matrice 600 Pro (SZ DJI Technology Co., Ltd. Shenzhen, China) and the Parrot Sequoia agriculture multispectral camera, which includes Green (G), Red (R), Red Edge (REG), and Near-infrared (NIR) bands, are part of the UAV image acquisition system (Table 1). During the UAV image acquisition period, the UAV’s flying height was set to 50 m, and the spatial resolution was set to 5 cm. Each flight trace had a 60% overlap ratio. After that, the UAV image and the associated GPS data were loaded into the Pix4D Mapper for preprocessing, which included geometric correction, radiometric calibration, and orthorectification.

### 2.3. Soil Sampling and Laboratory Procedures

One hundred and twenty sample sites and 40 ground control points were evenly distributed in the test area. An EC110 portable salinity meter equipped with a 2225FST series probe (in which the temperature correction for the electrical conductivity had already been completed) (Spectrum Technologies Inc., Dallas/Fort Worth, TX, USA) was used to make five measurements at and near each sampling site, with a range of no more than 5 cm × 5 cm. Using the five-point sampling approach, samples from 0–10 cm soil surface layer were taken at each survey location and put into separate sealed plastic bags. Meanwhile, the hand-held differential GPS (Trimble GEO 7X, Trimble Inc., Sunnyvale, CA, USA) was used to record the longitude and latitude coordinates of each sampling location, while the camera captured and recorded the surrounding environmental information.

Soil samples were treated in the laboratory for natural air drying at room temperature. Coarse fragments such as stones were discarded. All the soil samples were then physically milled, thoroughly mixed, sieved to obtain the fraction less than 2 mm (fine earth fraction), and packaged in separate bags for salt content analysis. The soil samples were processed into the soil solution at a soil-to-water ratio of 1:5 [26,27]. The soil conductivity value was measured using an EC110 conductivity meter, and 30 extracts were chosen at random to compute the matching soil total salt concentration [28]. Equation (1) depicts the conversion connection between soil total salt concentration and extraction solution conductivity in the studied region [4].

(1) $$S_{t}=2.180\times EC_{1:5}+0.727$$

where $S_{t}$ is the total salt content of the soil (g/kg), and $EC_{1:5}$ is the conductivity of soil extract (mS/cm) with a soil–water ratio of 1:5. $EC_{1:5}$ is used to calculate the total salt content of different soil samples without measuring the ion composition, as shown in Equation (1). For each treatment, the measurement was performed five times.

### 2.4. Construction of Soil Salinity Retrieval Index

The sensitive reflectance will be used to build a new soil salinity retrieval index. Before that, the correlation coefficient technique and grey correlation analysis between band reflectance and soil salinity content were primarily computed to screen sensitive band reflectance. The grey correlation analysis technique is a statistical analysis approach using several factors. It is used to calculate the degree of correlation among components based on the similarity or dissimilarity of development patterns among factors, i.e., the grey correlation degree [29]. The Pearson correlation coefficient measures the degree of linear association between two distance variables. The Pearson correlation analysis is a type of factor correlation analysis that is appropriate for continuous variables [30]. Besides, the band diagnostic index ($P_{i}$) was employed in this study to further improve the accuracy and reliability of screening sensitive band reflectance. The calculation equation of the band diagnostic index is shown below.

(2) $$P_{i}=R_{i}\times \sigma_{i}$$

where $R_{i}$ is the correlation coefficient between the reflectance value on each band and the soil salinity, and $\sigma_{i}$ is the standard deviation of reflectance value of band *i* [31].

### 2.5. Validation

To examine the performance of the new proposed index, six vegetation indexes, six salinity indexes, and one brightness index were used as comparisons to conduct screening, model construction, and validation process. The vegetation index is calculated using the standard multispectral RS bands R and NIR, and it includes the Normalized Difference Vegetation Index (NDVI), Difference Vegetation Index (DVI), Soil Adjusted Vegetation Index (SAVI), and Ratio Vegetation Index (RVI). Based on the band operation of the NDVI, the Green Normalized Difference Vegetation Index (GNDVI) and the Red Normalized Difference Vegetation Index (NDVI_REG_) were calculated and classed as VI indexes. The salinity index stands for the soil salinity index. It is represented by six algebras (SI-T, SI1, SI2, SI3, NDSI, and SRSI), with Soil Remote Sensing Index (SRSI) being the transformation and synthesis index of the Soil Salinity Index SI1 and the vegetation index NDVI (Table 2). The brightness index (BI) is determined using the R and NIR bands.

The soil salinity retrieval model and comparison techniques were constructed using QGIS, SPSS, and Matlab. Based on the newly constructed index and sensitive VI and SI, the retrieval models of soil salinity were built using the Partial Least Squares Regression (PLSR [37]), Multivariable Linear Regression (MLR [38]), Back Propagation Neural Network (BPNN [39]), Support Vector Machine (SVM [40]), and Random Forest (RF [41]) methods. The determination coefficient (*R^2^*), root mean square error (*RMSE*), and residual predictive deviation (*RPD*) were employed to evaluate the regression results. *R^2^* represents the consistency with which the model was established and validated. If *R^2^* is near to one, the model is more robust and has a better fitting degree. The *RMSE* is used to evaluate the model’s prediction performance. The lower the *RMSE*, the better the model’s prediction ability. The *RPD* is the ratio of the measured value’s standard deviation to the predicted error. When *RPD* is less than 1.4, the model cannot predict measured values; 1.4 ≤ *RPD* < 2 indicates that the model can roughly predict those values, and *RPD* more than or equal to 2.0 shows that the model has exceptional prediction ability. Models with high *R^2^* and *RPD* values perform better in terms of prediction and stability [42].

## 3. Results

### 3.1. Statistical Analysis of Soil Samples

The soil salt concentration varied from 0.264 to 20.651 g/kg throughout the test area, with an average of 7.583 g/kg and a standard deviation of 5.766 g/kg (Table 3). The salinity of the soil in the test area was typically high. Modeling set’s soil salinity varied from 0.277 to 20.675 g/kg, with an average of 7.575 g/kg and a standard deviation of 5.735 g/kg. Validation set’s soil salinity varied from 0.258 to 20.250 g/kg, with an average of 7.627 g/kg and a standard deviation of 5.864 g/kg. The mean and standard deviation of the modeling and validation sets are comparable to the statistical findings of all sample sets, which may decrease model creation and validation deviation in the latter stage and has modeling reliability.

### 3.2. Selection of Sensitive Bands

The correlation findings of UAV image reflectance showed that grey correlation coefficients have larger absolute values than Pearson correlation coefficients for the four-band reflectance and soil salinity content. Grey correlation coefficients between G, R, NIR, and salinity content were 0.567, 0.569, and 0.612, respectively, and were all significant at the 0.01 level (Table 4). Relative correlation coefficients were 0.532 (*p* < 0.01), 0.522 (*p* < 0.01), and 0.557 (*p* < 0.01) for G, R, NIR, and salinity content, which showed the same pattern as that of the grey correlation. Among the four bands, NIR had the greatest correlation coefficient.

To further improve the accuracy and reliability of screening sensitive band reflectance, the diagnostic index $P_{i}$ of G, R, REG, and NIR were computed. We can find that G, B, and NIR bands were higher than that of REG (Table 5), which further indicated that the soil reflectance of green, red, and near-infrared bands of UAV multispectral image were sensitive to soil salt information, which can be used to construct a sensitive soil salinity retrieval index.

### 3.3. Construction of Soil Salinity Retrieval Model

This study compared various combinations of the three-*soil* salinity sensitive bands (R, G, and NIR), e.g., addition, subtraction, and division (Table 6), and analyzed the relationship between these transformation indexes and soil salinity information. Finally, we devised a new index, namely the Soil Salinity Retrieval Index (SSRI, Equation (3)) to detect soil salinity by relying on the three sensitive bands.

(3) $$\mathrm{SSRI}=\frac{\mathrm{NIR}}{\sqrt{R*G}}$$

where G, R, and NIR is the green, red, and near-infrared band reflectance of the UAV image, respectively.

### 3.4. Correlation Analysis

The correlations of proposed SSRI, VIs, and SIs with soil salinity content were shown in Table 7. Among the 14 indexes, SSRI showed the higher grey correlation and Pearson correlation coefficients, 0.689 and 0.632, respectively. NDVI and DVI were the only two VIs that demonstrated a significant association (*p* < 0.01), with NDVI having the strongest correlation (0.619, 0.602). SI, SI-T, SI3, NDSI, and SRSI had a significant association with soil salinity (*p* < 0.01), with SRSI having the highest value of correlation (Table 7). Therefore, NDVI, SRSI, and SSRI were utilized to build soil salinity retrieval models.

### 3.5. Retrieval Accuracy

The RF, BPNN, SVM, PLSR, and MLR were used to create retrieval models of soil salinity based on the NDVI image. The results showed that the NDVI-based RF model showed the highest modeling and validation accuracies (*R^2^* = 0.625 and 0.633) among the five methods and then was BPNN, SVM, PLSR, and MLR in order of modeling and validation accuracies (Table 8). However, only the *RPD* of the RF model topped 1.4, which is the rough sample prediction threshold. Therefore, in the test area, NDVI is not suited for accurate soil salinity retrieval.

Table 9 displayed the statistically accurate findings of the five modeling approaches using SRSI. According to the statistical data, the accuracy of modeling and validation of the five modeling approaches is in the following order: RF > BPNN > PLSR > SVM > MLR. Except for the MLR model, the modeling and validation accuracy of the other four models are all more than 0.6. The vegetation index has the potential to extract soil salinity with acceptable accuracies.

In the test area, the *R^2^* values of RF, BPNN, SVM, PLSR, and MLR based on SSRI (Table 10) showed stronger fitting impacts than the retrieval model based on NDVI and SRSI (Table 8 and Table 9). Furthermore, the modeling and validation accuracies of the five techniques (RF, BPNN, SVM, PLSR, and MLR) were all higher than 0.6, and the *RPD* of the RF model is more than 2.2 (Table 10), which indicates that the RF has adequate soil salinity retrieval capacity.

**Figure 2:**
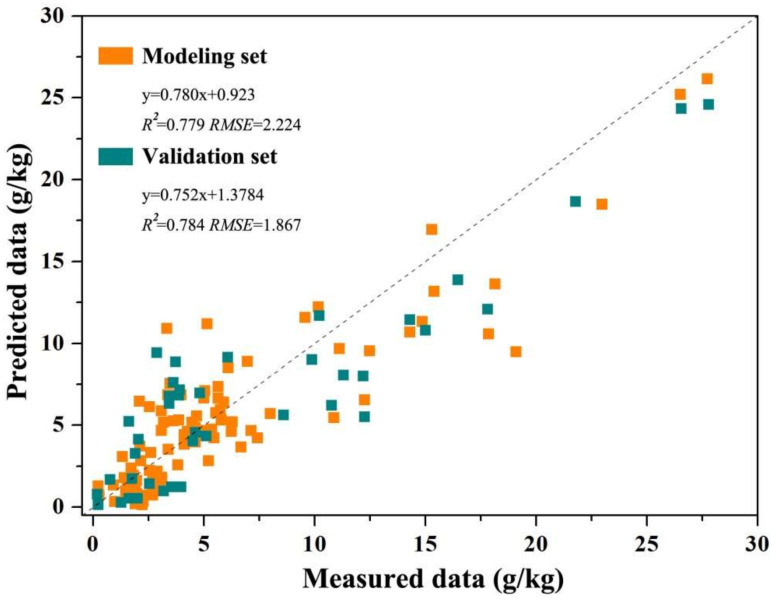
Scatter plot of the optimal retrieval model (SSRI-based RF method) of soil salinity based on UAV imagery.

The comparison of the modeling and validation accuracies (Table 8, Table 9 and Table 10) indicated that the retrieval models based on the proposed SSRI were more accurate than those based on vegetation index and soil salinity index. The soil salt retrieval modeling and validation accuracy were all greater than 0.638, and the *RPD* values were all greater than 1.463. Besides, among the five prediction modeling approaches, the order of modeling and validation accuracy was RF, BPNN, PLSR, SVM, and MLR. The modeling and validation accuracies of the RF modeling approach in various models were all greater than 0.6, and *RPD* values were above 1.424 (Table 8, Table 9 and Table 10). Among them, the *R^2^* and *RMSE* of the modeling set using the SSRI-based RF method were 0.724 and 1.746; and the *R^2^*, *RMSE*, and *RPD* of the validation set were 0.745, 1.879, and 2.211 (Figure 2), which were the highest. The optimal retrieval model of soil salinity in the test area is the SSRI-based RF method.

## 4. Discussions

The sensitive parameter and optimal retrieval method for soil salinity monitoring using UAV multispectral imagery were investigated in this study. The proposed soil salinity retrieval index (SSRI) based RF method was found to show the best accuracy in predicting soil salinity. The modeling *R^2^* and *RMSE* were 0.724 and 1.764, respectively; and the validation *R^2^*, *RMSE*, and *RPD* were 0.745, 1.879, and 2.211, respectively, which were the highest among all the models built using the five prediction approaches based on SSRI, vegetation index, and salinity index.

**Figure 3:**
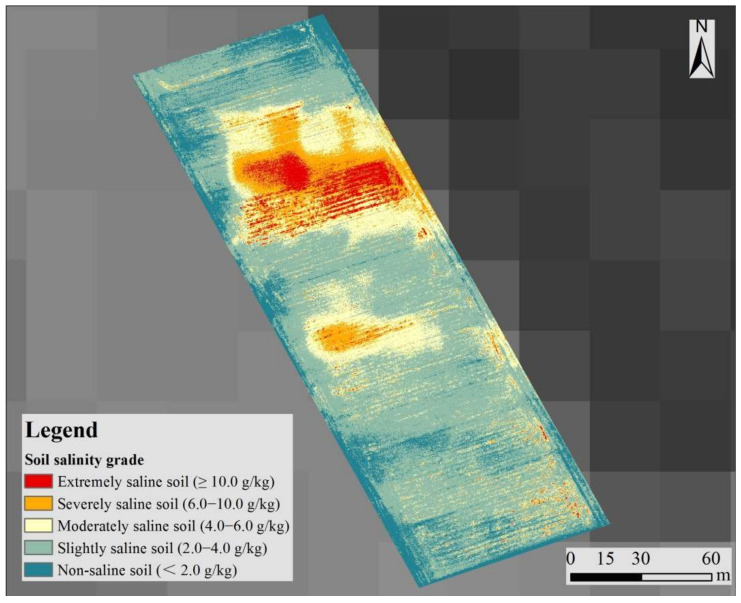
Retrieval map of soil salinity using the SRSI based RF method.

Compared to existing soil salinity retrieval studies using UAV imagery, this study screened sensitive band information and combined them to form a feasible index to help retrieve soil salinity. The retrieval values of soil salinity in the whole test area using the SSRI-based RF model (Figure 3) ranged from 0.323 to 21.210 g/kg, with an average value of 6.871 g/kg, which was close to the descriptive statistical results of the soil samples (Table 3). The test area can be divided into five grades based on the saline soil grading standard (Wang et al., 2019), namely extremely saline soil (salt content greater than 10.0 g/kg), severely saline soil (salt content 6.0–10.0 g/kg), moderately saline soil (salt content 4.0–6.0 g/kg), slightly saline soil (salt content 2.0–4.0 g/kg), and non-saline soil (Figure 3). According to the area statistical figures, the extremely saline soil occupied the lowest share of 5.3 percent of the five grades. Severely and moderately saline soil zones accounted for 15.5 and 13.6 percent of the overall test area, respectively. The proposal of slightly saline soil was 65.4 percent, the highest of the five categories. This pattern of soil salinity distribution is consistent with the observation in Figure 2, i.e., more than half of the sample locations were in the slightly saline region. The non-saline region encompassed 10.2 percent of the left test area. The geographical analysis demonstrated that soil salinization is widespread in the test area, with the majority of test sites belonging to the saline soil grade.

Visible and NIR bands displayed significant correlation links with soil salinity according to the results of two spectral screening analysis methodologies. The main minerals involved in the salinization of the soil of the YRD are rock salt and gypsum, with the main anions being Cl^−^ and SO_4_^2−^ and the main cations being Na^+^ and Ca^2+^ [11,43]. Previous research found that although NaCl has no spectral characteristics in the visible and near-infrared bands, NaCl is correlated with gypsum [44]. Gypsum possesses absorption qualities in the visible and near-infrared bands, which can help reveal soil salinity spectral information. Xu et al. (2018) found that gypsum has molecular vibration absorption spectrum features in the NIR band, visible and NIR band can collect SO_4_^2−^ spectral information [45]. Furthermore, studies have shown that salinized soil has higher reflectance in the visible and NIR bands than non-salinized soil [15,46]. Hence, spectral information of salinized soil retrieved from RS data can be used to estimate soil salinity in visible and near-infrared bands.

This study explored the sensitive parameters and optimal method to retrieve soil salinity, while soil samples were collected in the surface layer of soil (0–10 cm). For agriculture and food security, more attention should be paid to the indirect approach to a salinization assessment of root-zone (0–100 cm) [47]. Besides, the soil sample collection and measurement were conducted in one site. The proposed SSRI and the findings need more examination to test the reliability in further research. Furthermore, UAV multispectral image and the SSRI-based RF method can efficiently predict soil salinity with acceptable accuracy, whereas the UAV’s battery duration time prevents it from being used in large regional-scale soil salinity assessment. Recently, studies have fused satellite RS data with UAV images to derive regional-scale soil salinity, which is useful for estimating soil salinity across wide areas. However, it should be noted the variations in band wavelengths, meteorological conditions at the time of acquisition, and sensor compatibility between aviation and aerospace platforms are distinctly different. How to eliminate these uncertainties is a direction where further endeavors should be made in.

## 5. Conclusions

This study explored the sensitive parameter and optimal method for the accurate retrieval and spatial distribution of soil salinity. The sensitive band of soil salinity was discovered to be the band G, R, and NIR, a soil salinity retrieval index (SSRI) was proposed accordingly to retrieve soil salinity. SSRI-based RF method was the optimal combination that can accurately retrieve the soil salinity. Further study will be conducted in other salinized regions to examine the findings of this study.

## Figures and Tables

**Table 1:** Band information of multispectral camera sensor.

ID	Band	Abbreviation	Center Wavelength (nm)	Bandwidth (nm)
1	Green	G	550	40
2	Red	R	660	40
3	Red edge	REG	735	10
4	Near-infrared	NIR	790	40

**Table 2:** Spectral indexes and equations. G represents the reflectance of the green band, R denotes the reflectance of the red band, REG is the reflectance of the red edge band, and NIR is the reflectance of the near-infrared band.

Index Type	Spectral Index	Equation	Reference
VI	Normalized Difference Vegetation Index (NDVI)	$\frac{\mathrm{NIR}-R}{\mathrm{NIR}+R}$	[19]
	Difference Vegetation Index (DVI)	NIR−R	[19]
	Soil Adjusted Vegetation Index (SAVI)	$\frac{\left(1+L\right)\times \left(\mathrm{NIR}-R\right)}{\mathrm{NIR}+R+L},\;L=0.5$	[32]
	Ratio Vegetation Index (RVI)	$\frac{\mathrm{NIR}}{R}$	[32]
	Green Normalized Difference Vegetation Index (GNDVI)	$\frac{\mathrm{NIR}-G}{\mathrm{NIR}+G}$	[33]
	Red Normalized Vegetation Difference Index (NDVI_REG_)	$\frac{\mathrm{NIR}-\mathrm{REG}}{\mathrm{NIR}-\mathrm{REG}}$	[33]
SI	Salinity Index (SI-T)	$\frac{R}{\mathrm{NIR}}\times 100$	[34]
	Salinity Index 1 (SI1)	$\sqrt{\begin{array}{c} G\times \;R \end{array}}$	[34]
	Salinity Index 2 (SI2)	$\sqrt{\begin{array}{c} G^{2}{+R}^{2}{+\mathrm{NIR}}^{2} \end{array}}$	[35]
	Salinity Index 3 (SI3)	$\sqrt{\begin{array}{c} G^{2}{+R}^{2}\; \end{array}}$	[35]
	Normalized Difference Salinity Index (NDSI)	$\frac{R-\mathrm{NIR}}{R+\mathrm{NIR}}$	[36]
	Soil Remote Sensing Index (SRSI)	$\sqrt{{\left(\mathrm{NDVI}-1\right)}^{2}{+\mathrm{SI}1}^{2}}$	[32]
BI	Brightness index (BI)	$\sqrt{R^{2}{+\mathrm{NIR}}^{2}}$	[36]

**Table 3:** Statistics of soil salinity content.

Sample Set	Minimum (g/kg)	Maximum (g/kg)	Average (g/kg)	SD (g/kg)	Sample Size
All	0.264	20.651	7.583	5.766	120
Modeling set	0.277	20.675	7.575	5.735	90
Validation set	0.258	20.250	7.627	5.864	30

**Table 4:** Correlation analysis of sensitive reflectance with soil salinity.

Reflectance	Grey Correlation Coefficient	Pearson Correlation Coefficient
G	0.567 **	0.532 **
R	0.569 **	0.522 **
REG	0.550 *	S0.509 *
NIR	0.612 **	0.557 **

* Significant at 0.05 level, ** significant at 0.01 level.

**Table 5:** Diagnostic index of UAV image reflectance.

	G	R	REG	NIR
*R_i_*	0.567 **	0.569 **	0.550 *	0.612 **
*σ_i_*	0.791	0.761	0.470	0.732
*P* *_i_*	0.472	0.456	0.273	0.435

* Significant at 0.05 level, ** significant at 0.01 level.

**Table 6:** Equation combinations of the G, R, NIR.

ID	Algebra Operation
1	R+G+NIR
2	R-G-NIR, G-R-NIR, NIR-R-G
3	$\sqrt[{3}]{R*G*\mathrm{NIR}}$
4	$\frac{R}{\sqrt{G*\mathrm{NIR}}},\;\frac{G}{\sqrt{R*\mathrm{NIR}}},\;\frac{\mathrm{NIR}}{\sqrt{R*G}}$

**Table 7:** Correlation analysis of sensitive spectral index with soil salinity.

Spectral Index	Grey Correlation Coefficient	Pearson Correlation Coefficient
SSRI	0.689 **	0.632 **
NDVI	0.619 **	0.602 **
DVI	0.601 **	0.557 **
SRVI	0.512 *	0.476 *
RVI	0.517 *	0.458 *
GNDVI	0.557 **	0.514 *
NDVI_REG_	0.507 *	0.454
Salinity Index (SI-T)	0.607 **	0.559 **
Salinity Index 1 (SI1)	0.556 **	0.514 *
Salinity Index 2 (SI2)	−0.390	−0.200
Salinity Index 3 (SI3)	0.637 **	0.601**
NDSI	0.535 *	0.474*
SRSI	0.677 **	0.615**
Brightness Index (BI)	0.235	0.229

* Significant at 0.05 level, ** significant at 0.01 level.

**Table 8:** Accuracy statistics of the NDVI based retrieval model.

Modeling Method	Modeling Accuracy		Validation Accuracy		
	*R^2^*	*RMSE*	*R^2^*	*RMSE*	*RPD*
RF	0.625	2.977	0.633	2.789	1.425
BPNN	0.601	3.375	0.610	3.090	1.397
SVM	0.584	3.547	0.591	3.274	1.363
PLSR	0.557	3.645	0.566	3.455	1.321
MLR	0.492	3.988	0.488	4.714	0.670

**Table 9:** Accuracy statistical results of SRSI retrieval model.

Modeling Method	Modeling Accuracy		Validation Accuracy		
	*R^2^*	*RMSE*	*R^2^*	*RMSE*	*RPD*
RF	0.667	2.554	0.679	2.443	1.878
BPNN	0.641	2.631	0.653	2.781	1.750
SVM	0.619	3.205	0.621	3.029	1.549
PLSR	0.633	2.980	0.639	2.991	1.583
MLR	0.537	3.652	0.526	3.631	0.998

**Table 10:** Accuracy statistical results of soil salinity index retrieval model based on SSRI.

Modeling Method	Modeling Accuracy		Validation Accuracy		
	*R^2^*	*RMSE*	*R^2^*	*RMSE*	*RPD*
RF	0.724	1.764	0.745	1.879	2.211
BPNN	0.699	1.989	0.682	2.376	2.043
SVM	0.665	2.554	0.658	3.002	1.675
PLSR	0.671	2.275	0.689	2.897	1.748
MLR	0.639	3.091	0.622	2.994	1.464

## Data Availability

Data used in this study are available under request.
